# Prevalence, Pathology, and Alternative Control of Intestinal Parasites

**DOI:** 10.3390/pathogens14050433

**Published:** 2025-04-30

**Authors:** Jackson Victor de Araújo, Fábio Ribeiro Braga

**Affiliations:** 1 Departamento de Veterinária, Universidade Federal de Viçosa (UFV), Viçosa 36570-000, Brazil; 2 Departamento de Medicina Veterinária, Universidade Vila Velha, Vila Velha 29102-920, Brazil; fabio.braga@uvv.br

## 1. Introduction

Among the diseases that affect humans, there is a group that, due to their causes and consequences, continues to be part of the ‘unfinished’ agenda of single health: collectively, they are called neglected tropical diseases (NTDs). These diseases particularly affect poor and marginalised populations living in environments of generalised poverty, where resources are scarce. Among the NTDs, gastrointestinal parasites are caused by different types of agents, such as protozoa and helminths, which directly affect people’s health. Most of these parasites are highly neglected, such as the problems caused by protozoa that can infect various animal species and present a risk of zoonotic transmission. On the other hand, an ever-increasing exponential growth of people infected with helminths is estimated worldwide. Helminth infections are among the most common infections worldwide, with around 1.5 billion people infected, or 24 per cent of the world’s population [1,2]. On the other hand, there is still little data on the risk of infection in domestic animals, which could undoubtedly help to reduce the ‘scourge’ of helminthiases around the world, especially in underdeveloped countries [3].

However, it is important to mention that, in humans, the most diverse forms of environmental contamination and/or contamination by animal products can become the ‘gateway’ for these gastrointestinal parasites, which are often free in the external environment. In domestic animals, helminthic infections also cause damage that is often related to health, low productivity in herds and animal welfare. In companion animals (dogs and cats), gastrointestinal parasites often cause greater damage, especially in younger animals, and can be fatal.

Knowledge of parasite epidemiology is necessary for good planning to control and combat these infections, which have a direct impact on health. For example, the collection of epidemiological data can help identify the ‘installation and/or presence’ of certain gastrointestinal parasites, which possible hosts (definitive, paratenic and/or intermediate), whether they present a zoonotic risk, and contribute to the planning of healthy control measures. It should also be emphasised that the pre-parasitic forms of the most diverse helminths are present in the external environment, using their life cycle to reach the next stage (hosts) and thus promote disease.

With the exception of the use of ‘classic’ anti-parasitic drugs, which do not act completely on parasite cycles, more recent research has made some significant advances in the area, and could contribute directly to controlling these infections. On the other hand, the use of anthelmintic drugs remains the main strategy for controlling helminth infections in humans and animals. The administration of these drugs has made a significant contribution to reducing the prevalence of these infections in various regions of the world. However, inappropriate use, often without proper planning, raises concerns about the development of resistance and environmental contamination. Specifically, in relation to the environment, one of the biggest concerns is the impact of these drugs on non-target organisms, which can generate ecological imbalances and jeopardise efforts to effectively control helminth infections [2].

Therapeutic approaches based on epidemiological data, a reduction in the excessive use of medicines, and the implementation of appropriate sanitation practices may prove to be a good strategy associated with chemical control, although these strategies depend on the chemical group used, the dose administered and the particularities of each organism. In this sense, research ‘points’ to other advances in the area, such as biological control and its recent use of nanobiotechnology from nematophagous fungi, vaccines, resistant breeds, and the use of new herbal anthelmintics. These innovations are essential for a more effective future approach to controlling and combating these diseases. With regard to the use of nanobiotechnology in parasite control, research has demonstrated a variety of unique health applications, in particular bactericidal, antifungal and even antiviral efficacy, based on the use of silver nanoparticles produced by the helminthophagous fungus *Duddingtonia flagrans* (AgNPs) [4].

## 2. An Overview of the Published Articles

The growing awareness of One Health has led to a new global dynamic, with greater sustainability, food safety and an increasing search for animal welfare. The aim of this Special Issue was, therefore, to present the theme of ‘Prevalence, Pathology and Alternative Control of Intestinal Parasites’ in domestic animals and humans.

Contribution 1 (Ferraz et al.) evaluated the ovicidal activity of silver nanoparticles biosynthesised by the nematophagous fungus *D. flagrans* on *T. canis* eggs. The results showed destruction of up to 47% of the eggs and inhibition of development by 88% after 30 days, highlighting the potential of these nanoparticles in parasite control. Contribution 2 (Blanco-Costales et al.) compared the prevalence of anti-*Anisakis* antibodies in blood donors before and after the implementation of sanitary regulations. The results showed a significant reduction, especially in IgE levels (from 11.65 per cent to 2.2 per cent), indicating the effectiveness of preventive measures. Contribution 3 (Teixeira et al.) recorded a high prevalence of parasites in dogs (53%) and cats (53%) on the islands of São Miguel and Terceira. Infections by Ancylostomatidae, *Toxocara* spp. and *Cystoisospora* spp. stand out, as well as a significant prevalence of lungworms in cats (20.87%). Contribution 4 (Pérez-Anzúrez et al.) showed that the nematophagous fungus *Lecanicillium psalliotae* had high ovicidal (up to 99%) and larvicidal (up to 96.8%) efficacy against *Haemonchus contortus*, a haematophagous parasite of sheep. The presence of alkaloids and tannins in the liquid cultures of the fungus may be related to its anti-parasitic activity. Contribution 5 (Pilarczyk et al.) identified a prevalence of 61.8 per cent of intestinal parasites, including zoonotic species such as *Echinococcus multilocularis* (10.9 per cent), Alaria alata (17.6 per cent), and *Toxocara canis* (28.5 per cent) in foxes. Contribution 6 (Class et al.) isolated and genetically characterised B. coli from pigs on family farms in Brazil. Pavlova medium supplemented with foetal bovine serum was the most effective for isolation. Molecular analyses identified genetic variants of the protozoan. Contribution 7 (Ortega-Carballo et al.) tested Stevioside, a compound found in the plant Stevia rebaudiana, which showed amebicidal activity against *Entamoeba. histolytica*. A reduction in cell viability, morphological changes in trophozoites, and a decrease in the expression of cysteine proteases were observed, suggesting its potential as a therapeutic alternative. Contribution 8 (Arsenopoulos et al.) revealed a high genetic diversity of *Haemonchus* spp. in domestic ruminants in Greece, with multiple strains circulating in the country. These data are essential for the development of effective control and prevention strategies. In Contribution 9 (Araújo et al.), the authors carried out a review on the use of helminthophagous fungi, such as *Duddingtonia, Arthrobotrys* and *Pochonia*, in the biological control of zoonotic intestinal helminths. These fungi have proved effective in reducing environmental contamination by parasites such as *Toxocara, Ancylostoma*, and *Schistosoma*.

## 3. Conclusions

The various solutions presented here are of natural origin, and the use of biotechnological tools to document prevalence has become increasingly common. In this sense, new research, experimental designs, methods, and processes are being developed dynamically in research centres and universities, which creates a fruitful and largely positive environment. This Special Issue has brought together important contributions from researchers in different countries and continents, highlighting in particular the prevalence of the most distinct parasites and clearly demonstrating the rich diversity of scientific perspectives on the control of the parasites that plague so much of our health.
